# Knowledge, awareness, and perception on genetic testing for primary immunodeficiency disease among parents in Malaysia: a qualitative study

**DOI:** 10.3389/fimmu.2023.1308305

**Published:** 2024-01-12

**Authors:** Ahmad Hazim Syakir Ahmad Azahari, Farheen Hakim Zada, Intan Hakimah Ismail, Intan Juliana Abd Hamid, Bruce Wee Diong Lim, Noor Akmal Shareela Ismail, Adli Ali

**Affiliations:** ^1^ Department of Paediatrics, Faculty of Medicine, Universiti Kebangsaan Malaysia, Kuala Lumpur, Malaysia; ^2^ Clinical Immunology Unit, Department of Paediatrics, Faculty of Medicine and Health Sciences, Universiti Putra Malaysia, Serdang, Malaysia; ^3^ Primary Immunodeficiency Diseases Group, Department of Clinical Medicine, Institut Perubatan dan Pergigian Termaju, Universiti Sains Malaysia, Bertam, Pulau Pinang, Malaysia; ^4^ Malaysian Patient Organisation for Primary Immunodeficiencies (MyPOPI), Kuala Lumpur, Malaysia; ^5^ Hospital Tunku Ampuan Besar Tuanku Aishah Rohani, UKM Specialist Children’s Hospital, Universiti Kebangsaan Malaysia, Kuala Lumpur, Malaysia; ^6^ Department of Biochemistry, Faculty of Medicine, Universiti Kebangsaan Malaysia, Kuala Lumpur, Malaysia

**Keywords:** knowledge, awareness, perception, genetic testing, primary immunodeficiency disease, parents

## Abstract

**Background:**

Primary Immunodeficiency Disease (PID), also known as Inborn Errors of Immunity (IEI), comprises a group of rare genetic disorders that impair the body’s immune responses. These conditions result from monogenic germline mutations that affect the function of genes governing the innate and adaptive immune system. Therefore, individuals with PID are more susceptible to infectious diseases, allergies, and autoimmune and autoinflammatory conditions. The prevalence of PID has been on the rise, with the number of classified diseases reaching 404, and 430 genetic defects reported to cause these conditions. However, in Malaysia, genetic testing for PID is currently limited and needs to be outsourced to overseas laboratories, posing financial challenges for families. Moreover, limited research has focused on the knowledge and awareness of genetic testing among parents of children with PID in Malaysia. This study aims to address this gap and provide valuable insights into the knowledge, awareness, and perception of genetic testing among this specific population.

**Method:**

This qualitative cross-sectional study utilised online open-ended, semi-structured focus group interviews to explore the perceptions and experiences of parents of children with Primary Immunodeficiency (PID). Participants were recruited through convenience sampling from the Malaysian Patient Organisation for Primary Immunodeficiencies (MyPOPI), a non-governmental organisation dedicated to providing support and raising awareness about PID. The study spanned from May 2023 to July 2023 and included participants from diverse regions of Malaysia who had undergone different diagnostic journeys in various hospitals.

**Result:**

The focus group discussions yielded 11 sub-themes that highlighted the experiences, understanding and challenges of the participants regarding genetic testing based on the semi-structured questions. These sub-themes were then grouped into four main themes that are awareness and understanding of genetic testing, the journey towards diagnosis and treatment, emotional impact and psychological factors, and the importance of medical experts in diagnosing and managing PID, as well as public perception and awareness.

**Conclusion:**

In conclusion, this study highlights the diverse knowledge, awareness, and perception surrounding genetic testing for PID. Factors such as access to services, family history, and personal circumstances shape individuals’ understanding of genetic testing. The importance of healthcare professionals, along with the need for improved accessibility and targeted communication strategies, is underscored to enhance understanding and reduce stigma surrounding genetic testing for rare diseases like PID.

## 1 Introduction

Primary Immunodeficiency Disease (PID) or also known as Inborn Errors of Immunity (IEI), encompasses a group of rare genetic disorders leading to the impairment of the body’s immune responses. PIDs arise from monogenic germline mutations, resulting in loss of expression, loss-of-function, or gain-of-function from the encoded proteins. These variants of the functional genes that govern the innate and adaptive immune system cause immune defects, increasing susceptibility to infectious diseases, allergies, autoimmune, and autoinflammatory diseases (1). Previous publication from the International Union of Immunological Societies Expert Committee in 2019 has reported additional classification of PIDs into 404 diseases with 430 genetic defects linked to these conditions (1, 2). The current prevalence of PID is likely to increase by 10%, shifting from 1/10,000 – 1/50,000 births to 1/1,000 – 1/5,000 births (1, 3).

There is a wide range of clinical manifestation for patients with PID, ranging from increased susceptibility to infections such as recurrent pneumonia, sinus infections to significant immune dysregulation which often lead to multiple autoimmune manifestations, delayed growth and development, lymphoproliferation and malignancy (4–6). Early diagnosis of PID diagnosis is crucial, as it correlates with effective treatment delivery and improved survival outcomes. For instance, patients with severe combined immunodeficiency (SCID) face a grim prognosis if diagnosed too late, without receiving allogeneic hematopoietic stem cell transplantation; resulting in a survival rate beyond their first year (5, 7). In 2019, Abd Hamid and colleagues reported a prevalence rate of 0.37 cases per 100,000 population for PIDs in Malaysia. Their review of PID literature identified a total of 119 PID cases, with only 26 patients received molecular diagnosis.

Since the establishment of medical genetic services in Kuala Lumpur dated back in 1994, the overall aspect of genetic services has improved significantly with the availability of genetic counselling, genetic testing, and diagnosis for paediatric and adult-onset rare diseases (8). As important as other components of genetic services, genetic testing has emerged as a powerful tool for patients affected with rare diseases. It serves as a key instrument in concluding their diagnostic odyssey journey. Additionally, for clinicians, genetic testing enables the formulation of disease management plans and provides insights into available treatment options. Furthermore, leveraging current knowledge and technology, genetic testing can identify specific genetic variants associated with rare diseases through the analysis of an individual’s DNA, enabling a more accurate molecular diagnosis.

However, genetic testing while invaluable in patient-centered care, presents both advantages and challenges. In general, the potential benefits for genetic testing are (i) to provide molecular diagnosis and identify disease causing variants, (ii) for early detection and prevention of secondary conditions, (iii) to limit the need for more intrusive and expensive testing (9, 10), (iv) to come out with a management plan and treatment options (11), (v) to provide genetic risk assessment for family members and for future pregnancy planning (12) and most importantly (vi) to give autonomy to patients in making informed medical and lifestyle decisions (13). In contrast to that, there are also challenges associated with genetic testing which may involve ethical, legal, and social issues. These include psychological burden on parents and families, such as issues of blame and genetic guilt (14), genetic discrimination from the community (15), financial issues, and the uncertainty stemming from the identification of variants of uncertain significance (VUS) results (16). VUS is among the potential outcomes of a genetic test, and it fails to meet the criteria for classification as either pathogenic or benign, and the existing evidence for the variant is conflicting (17). With the progression of genetic testing methods, a larger number of genes are under analysis, leading to a rise in the identification of VUS results. Nonetheless, comprehending VUS proves to be complex since this outcome is not suitable for diagnostic purposes and does not influence medical management, where parents of children receiving VUS results often struggle to comprehend them, which can result in adverse outcomes such as feelings of frustration and helplessness (18).

The role of genetic testing in the diagnosis of numerous PID is significant. It can establish a conclusive diagnosis together with clinical and immunological information, predict prognosis through genotype-phenotype correlations, utilise targeted therapies, and guide decisions related to family planning. As part of future pregnancy planning, parents may be presented with the option of prenatal diagnosis or preimplantation genetic diagnosis (19–21). Another crucial component in the diagnosis of PID is the expert management provided by clinical immunologists. Unfortunately, the availability of clinical immunologists in Malaysia is insufficient, causing delays in the detection and diagnosis of PID cases. Furthermore, clinical immunology is not officially recognised by the National Specialist Register of Malaysia as a distinct subspecialty field. Consequently, patients encounter restricted access to such services. Within the Ministry of Health (MOH) in Malaysia, many PID patients are managed by other pediatric subspecialties, such as Pediatric Respiratory Specialists, based on their availability within MOH facilities (5).

Genetic testing for PID is often offered to individuals suspected of fulfilling the criteria for PID, mainly during childhood. Currently, this testing is not available in Malaysia and is outsourced to overseas labs, with families self-funding the expenses or seeking financial aid. This test is usually decided by the parents or legal guardians since children are unable to give their consent for genetic testing. Hence, this lies the importance of addressing the perception and awareness of genetic testing among parents of children affected by PID. To date, there is limited study focusing on the knowledge, awareness, and perception on genetic testing within the rare diseases community, such as PID, in Malaysia. Existing studies available primarily explore cancer genetics (22), autism spectrum disorder (ASD) (23), and hereditary disorders (24). To the best of our knowledge, this study represents the first attempt to examine parental perceptions of genetic testing for PID in Malaysia specifically.

## 2 Materials and methods

### 2.1 Study design, participants, and data collection

In this qualitative cross-sectional study, online open-ended, semi structured focus group interviews were conducted among parents of children with PID from May 2023 to July 2023. Participants were recruited using a convenience sampling method from Malaysian Patient Organisation for Primary Immunodeficiencies (MyPOPI). MyPOPI is a non-governmental organisation that has been providing support and raising awareness about PID in Malaysia. Individuals registered with the MyPOPI group are either patients or parents of patients who have been clinically or molecularly diagnosed with PID. The members in MyPOPI group come from all part of Malaysia and their diagnostic journeys vary across different hospitals, which is why it was chosen as part of this study. Currently, MyPOPI has approximately 120 registered members from the medical community, volunteers, family members and individuals affected. The approval of this study was granted by the Research and Ethics Committee of Universiti Kebangsaan Malaysia (Project Code: JEP-2022-800).

During the recruitment process, participants for this study were selected according to the specific eligibility criteria. The criteria include that the participant can understand and read either English or Malay (Malaysian national language), and they must be parents of children with PID. The status of genetic testing is not compulsory, and parents of clinically diagnosed children are eligible to participate in this study. In assessing the adequacy of purposive samples in our study, the required samples were determined upon achieving saturation of emerging themes. In this aspect, we decided to utilise a “code meaning” approach, in which the saturation is deemed achieved in the subsequent interview when no new aspects, dimensions or nuances are identified. As documented, the number usually ranges between 9 to 17 respondents or between 4 to 8 focus group discussions (25).

An information sheet regarding the study was provided to the participants and they were recruited after giving their informed consent. Participants were then allocated into separate groups, either mothers or fathers, to ensure a homogeneous background during the interviews. To avoid researcher bias, a moderator (F.H.Z.), independent of the research team with no prior relationship with the participants, facilitated the interviews and discussions. The moderator asked a series of questions provided in the guides, with the flexibility to pose additional questions for a more comprehensive understanding of the participants’ perspectives. The focus group discussion lasted for about 1.5 to 2 hours. The interview questions were based on a previously published study and were adapted from qualitative and quantitative study (**Table 1**) by (26, 27), and Chin & Tham (24). The questions were validated through phases of revisions and discussions with experts in the field. The questions were translated into Malay language, and most of the discussion was conducted in Malay or in bilingual (Malay and English), given that majority of the participants were from the Malay ethnicity.

**Table 1:** List of questions asked to focus group participants.

Interview questions for Focus Group Discussion
**Knowledge** 1. Can you please share with us your understanding/opinion about genetic testing and how it can be used to identify a specific disease? 2. What is your understanding/opinion on how genetic testing can identify the risk of inheriting a genetic disease that is running in a family or passing down to the next generation? (Do they know their carrier status or recurrence risk) 3. Genetic testing can be done during pregnancy to find out whether the baby will develop diseases, which is known as prenatal screening. What is your opinion on this? 4. What are the benefits of genetic testing that you have heard of? 5. Do you think the knowledge that you have on genetic testing is adequate?
**Awareness** 1. What is your opinion on genetic testing services availability in Malaysia and where can one seek genetic testing? 2. Genetic disorders are caused by changes in our body information and not all genetic disorders can be cured. Are you aware of this? 3. What is your opinion regarding the public’s view and awareness on genetic testing? 4. Do you encounter any problems when gaining knowledge on genetic testing? (Either online info or from doctors). 5. Do you think all genetic testing can provide an exact diagnosis? 6. Are you aware there are some commercial genetic testing kits available online and in pharmacies? Have you tried it before? (E.g., CircleDNA)
**Perception** 1. Do you think genetic testing is important? If yes or no, why? (Does it raise any ethical concern if no) 2. In your opinion, what are the factors involved when considering opting for genetic testing? Personally, would you consider genetic testing yourself? (Would you do it on yourself if you haven’t) 3. There is a common misconception that genetic testing always provides clear answers, what is your opinion on the reliability of genetic test results? Do you think it tampers with nature, religion, and their beliefs? 4. Do you think lack of education and knowledge on genetics and genetic tests are what raised ethical issues in genetic testing? 5. Is it important to raise awareness of genetic testing in Malaysia? What are the ways to implement government laws and policies to ensure the safe and effective use of genetic testing in Malaysia?

All participants recruited have or had a child diagnosed with PID and are registered members of the MyPOPI support group. A recruitment announcement was disseminated through the MyPOPI WhatsApp group. Additionally, more than thirty participants were contacted individually and invited to participate through WhatsApp for this focus group discussion.

### 2.2 Data analysis

All FGD sessions were recorded to ensure precise data collection with the permission of the participants, after which they were transcribed into Word documents. These transcriptions were then cross-verified against the digital recordings to confirm accuracy. The transcripts underwent multiple readings and were independently coded by the researcher (A.H.S.A.A.). Thematic analysis, following the approach outlined by Braun and Clarke (28), was analysed by the research group (A.H.S.A.A., A.A., N.A.S.I.). Based on coding analysis, the main and subthemes were identified. Final themes were decided and discussed until consensus was reached. Exemplar quotes were translated into English and presented in the results section.

## 3 Results

### 3.1 Description of participants

The study was conducted with a total of 15 parents that had provided consent. The demographic characteristics of the parents (interviewees) and patients’ characteristics include those of 12 mothers and three fathers (**Table 2**). Among those 15 participants, 13 had genetic testing done for their children and only seven participants had undergone genetic testing. Five participants who had undergone genetic testing were found to carry the same variants identified in their children while one participant did not carry the variant. Reasons for not undergoing genetic testing among other participants included cost factors, unwillingness to know the information, and not having the time yet to proceed with genetic testing.

**Table 2:** Description of the study participants.

Characteristics/*PN	PN 1	PN 2	PN 3	PN 4	PN 5	PN 6	PN 7	PN 8	PN 9	PN 10	PN 11	PN 12	PN 13	PN 14	PN 15
	FGD 1			FGD 2			FGD 3			FGD 4			FGD 5		
Gender															
Male										/	/	/			
Female	/	/	/	/	/	/	/	/	/				/	/	/
Ethnicity															
Malay	/	/	/	/		/	/	/		/	/	/	/	/	/
Chinese					/										
Indian									/						
Others															
Religion															
Islam	/	/	/	/		/	/	/		/	/	/	/	/	/
Buddhism															
Christian					/				/						
Hinduism															
**Age (Years)**	39	52	41	31	31	41	36	37	62	44	64	38	40	49	43
Education level															
Primary															
Secondary								/	/						
Tertiary	/	/	/	/	/	/	/			/	/	/	/	/	/
Household income															
RM1000 - RM5000			/	/				/		/	/	/	/		
RM5001-RM10000	/	/				/	/							/	/
>RM10001					/				/						
**Total number of children**	4	4	4	2	1	3	3	2	5	4	4	2	4	3	2
**Total number of children with PID and Diagnosis**	1 *SCID	1 *SCID	2 *WAS	2 *SCID	1 SCID	1 *XLA Brutons	2 *X. linked SCID	1 *XLA Brutons	1 *XLA Brutons	1 *Severe CN	1 *CVID	1 *XLA Brutons	1 *SCID	1 *SCID	1 *WAS
**Genetic testing done for patient**	Yes	No	Yes	Yes	Yes	Yes	Yes	Yes	Yes	Yes	No	Yes	Yes	Yes	Yes

*PN, participant number; XLA Brutons, X-linked agammaglobulinemia; WAS, Wiskott-Aldrich syndrome; SCID, Severe Combined Immunodeficiency Disease; CVID, Common Variable Immunodeficiency; Severe CN, Severe Congenital Neutropenia.

### 3.2 Thematic analysis

The focus group discussions yielded 11 sub-themes that highlighted the experience, understanding and challenges of the participants on genetic testing based on the semi-structured questions. The sub-themes were then grouped into four main themes. The main themes revolve around their knowledge and understanding on genetic testing, awareness on genetic testing and services, perception towards genetic testing and challenges in the molecular diagnosis of PID (**Table 3**).

**Table 3:** Quotes, sub-themes, and themes identified in this study through the analysis of interview transcripts.

Themes	Sub-themes	Examples of the Quotes
Knowledge and understanding on genetic testing	Genetic testing as a detection tool for PID	…our blood will be taken, and the blood is tested (*genetic test*) to detect if the individuals have certain disease *(genetic disease).* (PN1) … as far I remember, they (*doctor*) took blood and send it for testing. (PN12) … regarding genetic testing, I feel that I understand it now and I am capable of handling it. However, currently, it seems that only the patients themselves truly comprehend the situation (*difficult to explain to others*). (PN5)
	Indication of family history and personal circumstances to proceed with genetic testing	…if we have a family history of genetic disease, then it will be easier to proceed with genetic testing. (PN2) …when they know about the genetic (*molecular diagnosis result*), they quickly sent for testing and within one month they can make confirmation of the diagnosis. (PN3) …previously if you say that this condition is genetic (*inherited*) and that it runs in my family, I will believe less but not long after my nephew was born and he had the same condition (*frequent infection*), then I come to realise that this is indeed genetic (*the condition can be inherited*). (PN8)
	Benefits and importance of genetic testing	… the importance of providing genetic testing among pregnant mothers is crucial because it can give (*parents*) the emotional support and preparation they needed to face any potential genetic issues that may arise in their unborn child. (PN1) … So, when we know there is a genetic issue, the treatment (*IVIG or BMT*) can be initiated more quickly. The chances of the child recovering are greater. (PN7)
Awareness on genetic testing and services	Awareness about genetic testing and rare diseases	…when I first heard about genetic testing, I also don’t know what it is actually. (PN1) …public awareness on genetic testing is very low, moreover, for Malay community they believe in superstition saying that we did something wrong. (PN2) …even at my workplace, my boss would say, “If you already know that your child will face the same challenges (*affected with PID*), why continue (t*he pregnancy*)?” (PN3)
	Access to genetic services in Malaysia	… for genetic testing, we know that in Malaysia, it is not available in any health clinics. (PN1) … I don’t know where I can find this genetic service because every genetic test is arranged by the doctors for my son. For example, the first time at Sunway Medical Center (*private hospital in Malaysia*), the doctor suspected that my son had an immune system problem, so they promptly sent his blood sample to their lab in the US. (PN5) …okay honestly if focusing on genetic testing services I do not know where to go *(genetic clinic).* (PN7) …the second one (*second child affected with PID*) is because we wanted it to be fast. So, we sent it to the USA, there was a fee that we paid. I don’t remember how much. (PN7) …I know that they will send the test (*genetic test*) locally in Shah Alam (*Molecular genetic lab in Malaysia*) (PN8)
	Access and information sources on genetic disease and genetic testing	…I prefer both ways, whether it’s obtaining information online or through doctors. (PN5) … but I find it difficult to understand (*genetic test and PID*), so I keep asking the doctors and they say that if I want more information, I should also try Googling it. (PN8) … Initially, we relied solely on the doctors for information, but at that time, we didn’t have internet access. So, we turned to our friends who had access to Google and printed out the information (*genetic disease*) for us. (PN9)
Perception towards genetic testing	Genetic testing can help parents to be more prepared in the future and for the next generation	…prenatal testing is important for us because it can give emotional support and to prepare for the pregnancy if it is affected. (PN1) …my close family members, they understand about my child condition, and they had experienced a lot (*with the patient*), so their immense support made me strong. (PN3) …I wanted to try for another pregnancy hoping that it will be a baby boy, but my husband does not want to have any more children. (PN6) … I have a daughter who is not yet married so I told her that she needs to explain her condition to her partner so that they don’t blame anyone. (PN2) In my opinion, sharing knowledge and experiences (*on genetic disease*) can be helpful to some extent. However, I also have concerns about sharing too much, as it may instill fear in others. We tend to share such information within our close family members, as we fear that others (*extended family or public*) may become anxious if they don’t ask me directly. (PN7) Parents can make decision whether to have more children or not. (PN11)
	Reliability of genetic testing	…I am still wondering if my late child was really SCID or not because he did not have the symptoms like any other SCID children…and we cannot trace his file to see the report. (PN3) …we need to put effort, we cannot give up just like that. (PN3) Only by undergoing a genetic test can we truly identify the actual disease, and only after knowing the real disease can we determine the appropriate treatment. (PN4) That’s all we have. So, we believe because we believe, and that’s all we can believe, right? So, we leave it to the experts, even though they may not be 100% accurate, but at least they can be trusted. That’s the only way we can trust. (PN6) …it is difficult to say that genetic testing can provide exact diagnosis. Even if we ask the doctors, they say they would only know the tip of the iceberg only. It depends on the disease if it is well known then we can get a diagnosis. Sometimes, we cannot get an answer (*from genetic testing*). (PN11)
Challenges in molecular diagnosis of PID	Delayed in diagnosis, underwent multiple tests, and uncertainty	During my first child, I felt that it took a long time to confirm and detect the disease (*SCID*). But according to the doctor in the Alor Setar, Kedah area, they mentioned that now there are new specialists who are focusing on PID, specifically the disease related to PID. (PN2) …but it was his (*child*) of luck when we get to meet the right doctor who specialises in that specific field (*PID*)…(PN8)
	Difficulties to interact with medical doctors in government hospital	…even in the hospital if we tell them about SCID, they don’t understand, then who are we to explain to the specialist…imagine O&G specialist told me that SCID was not a genetic disease. (PN2) …but back then, when they kept changing doctors (*during clinic session*), each doctor would have a different perspective. One doctor would say one thing, while another would say something else. As the mother, I felt conflicted by these differing opinions. (PN3) …I think the awareness with the doctors is important. (PN9) … in Malaysia, when we go to the hospital, we always seen by different doctors, they (*doctors*) keep changing so it is difficult for us to discuss this kind of things (PID). (PN11)
	Cost factors	…they want to send my blood sample to see if I am a carrier, but the test needs to be sent to Australia and will cost around RM30k, so for me it is very much impossible, and I keep quiet for over 26 years. (PN2) …we want to focus our financial for our child and if I just want to know my carrier status, it cannot solve my child condition. (PN3) … I am not aware about the cost and was not informed about it either (*cost for genetic testing*). (PN10)

#### 3.2.1 Knowledge and understanding on genetic testing

During the discussions, one of the topics that emerged was the participants’ comprehension of genetic testing. It was found that the majority of the participants’ children had undergone molecular genetic testing to verify the diagnosis of PID. However, two patients were clinically diagnosed with SCID and CVID. Next, we identified the participants’ comprehension of genetic testing, including its process, and the circumstances in which it is recommended, particularly in cases involving a family history. The participants emphasised the advantages and importance of genetic testing, further prompting discussion on these aspects. This theme is further divided into three sub-themes:

##### 3.2.1.1 Genetic testing as a detection tool for PID

Throughout the focus group sessions, participants expressed and shared their knowledge regarding genetic testing. Surprisingly, most of them were unaware of genetic testing before undergoing the test. Their interactions with medical experts specialising in PID provided insights into the world of genetic testing. Participants came to understand that genetic testing usually involves the collection of a blood sample, which is then sent to laboratories located abroad for analysis. One parent expressed understanding about genetic testing for detecting PID but found it difficult to explain the test to others.

##### 3.2.1.2 Indication of family history and personal circumstances to proceed with genetic testing

Most participants reached a consensus that genetic testing may be justified when there is a positive family history of recurrent infections and frequent hospitalisations. They emphasised the importance of communicating this information to doctors if they are unaware, enabling healthcare professionals to be properly informed and adjust their management accordingly. Additionally, being aware of a positive family history can assist in assessing risks during pregnancy and help avoid administering vaccines that may exacerbate the patient’s condition.

##### 3.2.1.3 Benefits and importance of genetic testing

The participants underscored the significant role of genetic testing in facilitating early preparation for the arrival of a child, particularly during the prenatal stage. They recognised that early diagnosis through genetic testing enables timely and precise treatment, offering the opportunity to initiate interventions at the earliest possible stage. Additionally, the participants shared the belief that genetic testing can aid in both physical and mental preparations for their children. One mother, whose child had SCID, a severe combined immunodeficiency disorder, highlighted the resilience and determination of SCID mothers, who refuse to succumb to the disease or consider pregnancy termination. With effective treatments available for SCID, these mothers choose to fight for the necessary medical interventions. Overall, most participants agreed that they consent to genetic testing in the hope of finding answers to their children’s condition and receiving appropriate treatment.

#### 3.2.2 Awareness on genetic testing and services

The second theme explores families and the public’s awareness of genetic testing and services. Three sub-themes covered are as follows.

##### 3.2.2.1 Awareness about genetic testing and rare diseases

The awareness of genetic testing among the participants before having a child with genetic disease was lower compared to the present, where they have become more exposed to genetic testing. There is also a lack of public awareness regarding genetic testing for PID, resulting in limited understanding and knowledge about the benefits and implications of genetic testing for PID within the general population.

##### 3.2.2.2 Access to genetic services in Malaysia

The participants had limited knowledge about the available options for genetic testing. Their understanding primarily stemmed from interactions with clinical immunologists who facilitated the diagnosis of their children. In cases where family members expressed interest in genetic testing, participants would advise the family members to consult these clinical immunologists. While some participants were aware of local genetic testing facilities, they acknowledged that sending samples overseas could yield faster and more precise results. One mother expressed appreciation for the presence of a clinical geneticist at the hospital where she gave birth, as the geneticist provided valuable explanations about the testing process during her hospital stay.

##### 3.2.2.3 Access and information sources on PID and genetic test

Participants gathered information from various sources, including online resources and consultations with medical experts. They actively sought knowledge through online platforms, such as reputable websites and articles, to supplement their understanding of genetic testing. Additionally, they sought guidance from medical professionals who provided valuable insights, explanations, and personalised recommendations based on their expertise in the field of PID.

#### 3.2.3 Perception towards genetic testing

The analysis of participants’ responses revealed a positive perception towards genetic testing. This optimism primarily stems from the recognition that genetic testing equips parents with improved preparedness in addressing their children’s health concerns. Participants indicated that genetic testing enhances their ability to communicate effectively with their children regarding diagnoses, enabling open and informed conversations about potential health outcomes. Furthermore, participants expressed that genetic testing plays a pivotal role in facilitating family planning decisions and future pregnancy plans. This comprehensive and favourable perspective underlines the multifaceted advantages associated with genetic testing, ranging from informed parenting to making well-considered decisions about family dynamics and future healthcare choices. Two subthemes covered in this theme:

##### 3.2.3.1 Genetic testing can help parents to be more prepared in the future and for the next generation

The results underscore the significant role of genetic testing in enhancing parental preparedness for the future, both for their immediate family and subsequent generations. Participants consistently emphasised that genetic testing empowers parents with valuable insights into potential health risks and conditions that may impact their children and future generations. Parents have the tools and information to make smart choices about caring for their family’s health. With genetic testing, they can learn about potential health issues that could affect their children and even future generations. This helps parents to plan, take early action, and make lifestyle changes that contribute to their family’s well-being. Genetic testing serves as a means to be ready and informed, ensuring that future generations have the necessary tools to handle any health challenges that might arise.

##### 3.2.3.2 Reliability of genetic testing

The majority of participants expressed confidence in the reliability of genetic testing. For some, the journey to diagnose their children had been filled with uncertainty and confusion, leading them to believe that genetic testing could offer definitive answers. They saw genetic testing as their only hope to bring an end to the long and challenging diagnostic journey, and thus entrusted their faith in this test. In situations where uncertainty persisted, they were willing to leave the outcome to fate, understanding that genetic testing provided the best opportunity for clarity and resolution.

#### 3.2.4 Challenges in the molecular diagnosis of PID

The study highlighted several challenges associated with the molecular diagnosis of PID. Participants commonly reported experiencing delays in diagnosis due to the necessity for multiple tests and lingering uncertainties. Additionally, participants expressed difficulties in effectively communicating and collaborating with medical professionals in government hospitals, which could potentially impede the diagnostic process. Furthermore, cost emerged as a notable challenge, as the financial burden of molecular diagnostic procedures acted as a deterrent for some participants. These challenges collectively underscore the multifaceted nature of obstacles faced in the molecular diagnosis of PID, encompassing aspects of procedural complexity, healthcare system interactions, and economic considerations.

##### 3.2.4.1 Delayed diagnosis, undergone multiple tests, and uncertainty

Participants expressed frustration with the lengthy diagnostic process for their children, which involved multiple clinical tests, including an HIV test. They also faced legal issues due to false accusations of rape due to an ulcer in the child’s anus. Concerns about frequent hospital visits and the mental toll of not being able to undergo prenatal testing were additional challenges. These challenges highlighted the participants’ desire for comprehensive genetic testing and timely interventions.

##### 3.2.4.2 Difficulties to interact with medical doctors in government hospital

The study found that patients often struggled to communicate with medical doctors in government hospitals due to frequent change of doctors, making it hard to have consistent conversations about their disease. Another significant problem was the limited knowledge of many doctors and specialists in these hospitals regarding rare diseases like PID. This lack of knowledge posed difficulties for patients in obtaining the right information and help they needed. These issues highlight the challenges patients face in talking to doctors and receiving proper care in government hospitals, especially when there is a lack of continuous care and awareness about rare diseases.

##### 3.2.4.3 Cost factors

Participants acknowledge that the cost can pose a challenge when it comes to genetic testing. They note that most tests are not sponsored, requiring them to personally cover the expenses. In the past, the price of genetic tests was exorbitant. Additionally, participants express the desire to prioritise saving money for the care of their affected child with PID.

## 4 Discussion

Genetic testing in Malaysia has been advancing, yet there is a notable gap in comprehensive studies addressing the knowledge, awareness, and perception regarding genetic testing, particularly concerning Primary Immunodeficiency Disease (PID). While Malaysia is making strides in genetic research and healthcare, a thorough examination of public and healthcare professionals’ understanding and perspectives regarding genetic testing is essential for informed healthcare decisions. Specific to PID, a dedicated study evaluating the knowledge and awareness of the families of children with PID would bridge the gaps and opportunities in the diagnosis and management of this group of rare genetic disorders, potentially leading to improved healthcare outcomes and enhanced genetic services in the country.

The findings of our study revealed an adequate level of awareness and understanding among participants regarding genetic testing for PID when their child was first suspected of having PID. Most participants showed improvement in knowledge and understanding of genetic testing for PID after first hearing about it and conducting further research. However, it was observed that a few participants still had a low understanding of genetic testing even after experiencing it. Some issues were raised by participants with a low understanding, believing that since only one child in the family was affected with PID, there was no inherited risk. In some cases, no specific genetic diagnosis was found. This led to confusion about whether the illness in their child was genuinely inherited or simply a random occurrence. The performance of genetic testing for PID faced obstacles concerning expenses, availability, and understanding of results. Interpreting results can be challenging due to recent advancements in identifying variations linked to PID. It is crucial to note that not every variant result in a disease, and making such assertions requires solid evidence and thorough research. Understanding genetic test results involves connecting a positive outcome with the actual disease characteristics (19). In addition, some participants demonstrated a good understanding of genetics and genetic testing, while others had limited knowledge in this area. This disparity in awareness can be attributed to differences in educational background and exposure to genetic information. Participants with a better understanding of genetics were more likely to recognise the benefits and importance of genetic testing for PID (29).

In the context of awareness, family history and personal circumstances with PID play an important role in shaping individuals’ awareness and perception of genetic testing. Participants with a family history of PID are more likely to be aware of the importance of genetic testing and the potential benefits it offers in terms of early diagnosis and appropriate treatment. On the other hand, those without a family history of PID often had limited awareness, a sense of denial towards the diagnosis and they would rely on other sources for information on the disease (30). Apart from having the strongest warning signs of PID which is a family history, knowing the signs and symptoms of PID is also important in the process of recognising the disease (31).

Access to genetic services emerged as a significant factor influencing awareness and understanding of genetic testing. Participants who had easy access to genetic services, such as genetic counsellors or specialised clinics, demonstrated a higher level of knowledge and understanding of genetic testing. However, individuals from remote areas or with limited healthcare resources faced challenges in accessing genetic services, which affected their awareness and understanding of genetic testing for PID (32). In this study, most participants had little knowledge about the access to genetic services in Malaysia, as their child was being treated by a clinical immunologist at the time of diagnosis. In addition, access to information sources may help the patient in the diagnostic journey and understanding of genetic testing. Participants who had access to reliable and up-to-date information sources, such as specialised healthcare centers or patient support groups, were able to navigate the diagnostic process more efficiently. However, individuals who lacked access to such information sources faced difficulties in obtaining accurate information about PID and genetic testing, leading to further delays in diagnosis.

The role of clinical immunologists in diagnosing and treating PID was identified in our study. The participants emphasised the necessity of healthcare providers who possess comprehensive knowledge and understanding of PID and genetic testing. Those who had positive encounters with well-informed healthcare professionals reported greater satisfaction and confidence in their diagnosis and treatment plans. These findings highlighted the crucial role of knowledgeable medical experts in effectively managing PID (5) and the need to increase the number of clinical immunologists per population in Southeast Asia (20). Correspondingly, families of PID patients may rely on the healthcare provider administering treatment to acquire knowledge and understanding regarding genetic testing. However, according to an unpublished study conducted among healthcare providers in semi-government hospitals, a significant majority of the study population exhibit inadequate knowledge and awareness regarding the use of genetic testing in PID management. This study concluded that insufficient education and hands-on experience in utilising genetic testing for PID management contribute to the lack of awareness among the study population on genetic testing. Hence, it is essential to increase awareness initiatives in society about PID, the accessibility of genetic testing, and the crucial role of genetic healthcare professionals in establishing capable and secure healthcare environments in Malaysia (Saain et al., 2022)^1^.

Correlates to the previous statement, poor interaction with healthcare professionals were brought up by some participants, leading to frustration and dissatisfaction throughout the diagnostic journey. Insufficient communication, lack of empathy, and limited knowledge on the part of healthcare professionals contributed to negative experiences and hindered the diagnostic process for PID. Enhancing the knowledge and understanding of healthcare professionals through continuing education and specialised training in genetic testing for PID is crucial in improving patient experiences and outcomes (33, 34).

Additionally, the participants expressed that there is limited public awareness about genetic testing and rare diseases, including PID. Participants indicated that genetic testing and rare diseases are not well understood or recognised by the public. This lack of awareness can lead to misconceptions and stigmatisation surrounding genetic testing and PID, further hindering access to appropriate healthcare services and support. In a recent 2020 study on genetic testing for hereditary disorders in Malaysia, it is found that there is a positive perception on genetic testing among 450 respondents that reside in Klang Valley (24). Further research is required to assess public opinions regarding genetic testing for rare diseases, aiming to enhance the accessibility of genetic testing services, reducing the stigma in genetic testing and early disease detection (35).

Communication emerged as a key factor in raising public awareness about genetic testing and rare diseases. Effective communication strategies, including public health campaigns, educational initiatives, and media engagement, can play a significant role in increasing awareness and understanding among the public. Collaborative efforts between healthcare professionals, patient advocacy groups, and policymakers are needed to bridge the gap in public awareness and ensure that individuals have accurate information about genetic testing for PID (36). The statement PN7 highlights the potential benefits and concerns of sharing knowledge and experiences regarding genetic diseases. While sharing such information can be beneficial to some extent, there is a worry that excessive sharing may create unnecessary fear or anxiety in others. Consequently, individuals tend to restrict the sharing of such information to their immediate family members, as they fear causing distress to extended family or the public who may not directly inquire about it (37).

The emotional impact and psychological factors associated with genetic testing for PID were evident in our study. Participants highlighted the importance of support and preparation for children with PID when considering genetic testing. Many individuals expressed feelings of anxiety and fear, anticipating the potential results and implications of genetic testing. Genetic counsellors and healthcare professionals were seen as valuable sources of support in managing these emotional concerns and providing individuals with the necessary information and resources to cope with the testing process (38). Coping and acceptance were identified as important factors in the psychological well-being of individuals undergoing genetic testing for PID. Participants reported a range of coping strategies, including seeking emotional support from family and friends, engaging in informational and emotional support groups, and accessing mental health services. Acceptance of the test results, whether positive or negative, was found to be a significant factor in the emotional well-being of individuals and their ability to adapt to the implications of genetic testing (39).

Moreover, pursuing genetic testing can present various challenges for individuals. One primary challenge mentioned by the participants in the diagnostic journey is the financial aspect, as genetic testing can be costly, and insurance coverage may be limited or unavailable. Not all individuals have the financial means to afford these tests. Additionally, there may be limited accessibility to genetic testing facilities, particularly in rural or underserved areas, leading to geographical barriers. The lack of infrastructure needed for genetic testing locally in Malaysia is a significant barrier that restricts patients from accessing affordable testing (40). The psychological impact of genetic testing, such as receiving unexpected results or confronting the potential for genetic conditions, can also pose emotional challenges. Furthermore, ethical considerations, such as concerns about privacy and the potential for discrimination based on genetic information, can add complexity to the decision-making process of pursuing genetic testing (19, 41).

## 5 Limitation

The present qualitative study faced several limitations. Most participants were mothers of children with PID, and there was a lack of representation from fathers. This may have limited the diversity and representativeness of the finding. The low participation of fathers’ perspectives could affect the comprehensiveness of insights, as their unique perspectives and experiences might not be adequately represented. These limitations should be considered when interpreting the results and generalising the findings to other populations or contexts.

## 6 Conclusion

In conclusion, this study highlights adequate knowledge, awareness, and a positive perception surrounding genetic testing for PID. Factors such as access to genetic services, family history, and personal circumstances can influence individuals’ understanding and awareness of genetic testing. The diagnostic journey for PID is often characterised by delayed diagnosis, multiple tests, and high-cost factors, emphasising the need for improved accessibility and information sources to parents on genetic testing. Emotional impact and psychological factors play a significant role in individuals’ experiences with genetic testing, underscoring the importance of support and coping strategies. The role of knowledgeable healthcare professionals in diagnosing and managing PID cannot be overstated, while public perception and awareness of genetic testing and rare diseases remain limited, calling for targeted communication strategies to enhance understanding and reduce stigma.

## Data availability statement

The original contributions presented in the study are included in the article/supplementary material. Further inquiries can be directed to the corresponding author.

## Ethics statement

The studies involving humans were approved by Research and Ethics Committee of Universiti Kebangsaan Malaysia. The studies were conducted in accordance with the local legislation and institutional requirements. The participants provided their written informed consent to participate in this study.

## Author contributions

AHA: Conceptualization, Data curation, Formal analysis, Investigation, Methodology, Writing – original draft. FH: Conceptualization, Formal analysis, Methodology, Writing – review & editing. II: Resources, Writing – review & editing. IA: Resources, Writing – review & editing. LB: Resources, Writing – review & editing. NI: Conceptualization, Formal analysis, Methodology, Supervision, Validation, Writing – review & editing. AA: Conceptualization, Data curation, Formal analysis, Investigation, Methodology, Project administration, Resources, Supervision, Validation, Writing – review & editing.

## Footnotes

1 Saain NF, Ali A, Ali A, Zahedi FD, Syed Omar SA. (2022). Knowledge, awareness, and practice of genetic on management of Primary Immunodeficiency Disorders (PID) [Unpublished manuscript]. Universiti Kebangsaan Malaysia (2022).
